# Results of the Adult COVID-19 Lifestyle Matching Study

**DOI:** 10.3389/ijph.2022.1604329

**Published:** 2022-02-18

**Authors:** Rui Zhong, Qiong Zhang, Yanfang Qiu, Lingxia Chen, Jianghua Xie, Yongjun Chen, Yajiao Zou, Lei Zhu, Li Tong, Yanhui Zou, Wei Wang, Yuhua Zhou

**Affiliations:** ^1^ Hunan Cancer Hospital/The Affiliated Cancer Hospital of Xiangya School of Medicine, Central South University, Changsha, China; ^2^ Yueyang Central Hospital, Yueyang, China; ^3^ Key Laboratory for Molecular Radiation Oncology of Hunan Province, Xiangya Hospital, Hunan, China; ^4^ School of Nursing, Hunan University of Chinese Medicine, Changsha, China; ^5^ Affiliated Nanhua Hospital, University of South China, Hengyang, China; ^6^ The First People’s Hospital of Changde City, Changde, China; ^7^ Yueyang City Junshan District the People Hospital, Yueyang, China

**Keywords:** smoking, COVID-19, coronavirus, SARS-CoV-2, comorbidity, lifestyle, Alcohol Consumption, betel quid chewing

## Abstract

**Objective:** The aim of our case-control study was to find the influence of lifestyle and comorbidities on COVID-19 susceptibility, identify risk factors and protective factors, and identify ways to encourage people to adopt a healthy lifestyle.

**Methods:** Patients with COVID-19 were matched with non-COVID-19 participants in a ratio of 1:2. Univariate analysis was performed using the chi-square test, and multivariate analysis was performed using conditional logistic regression.

**Results:** Multivariate analysis using conditional logistic regression found that alcohol consumption (AC) and a bland diet increased the risk of COVID-19, while college degrees and above, smoking, drinking tea, and exercise, especially walking, significantly reduced the risk of COVID-19.

**Conclusion:** After removing the effects of demographic factors, the study demonstrated that AC significantly reduced the ability of the body to resist COVID-19 infection. Moreover, following a bland diet increased the susceptibility to COVID-19. Notably, people who drank tea and performed regular exercises, especially walking, were significantly less likely to be infected with COVID-19. College degree or above relative illiteracy is COVID-19 protective factors of infection.

## Introduction

Coronavirus disease 2019 (COVID-19) is a respiratory disease caused by the SARS-CoV-2 virus. As of May 30, 2021, COVID-19 has affected nearly 170 million people and resulted in nearly 3.5 million deaths [1]. The immune system is the main entity that defends the body against viral infections, including COVID-19. Since it is still unclear why only some individuals are getting affected with COVID-19, prevention of COVID-19 is essential. Healthy lifestyles have been known to reduce the risk of multiple chronic diseases, including cancer, cardiovascular disease, and diabetes mellitus (DM), and to extend disease-free life expectancy [2, 3]. Moreover, studies have shown that regular exercise can prevent bacterial and viral infections and enhance the immune functions of the human body [4, 5]. Food and nutrition have also been shown to be factors affecting the function of the immune system, and many metabolic or chronic diseases are associated with poor dietary habits [6]. Sleep plays a great role in the develop and progression of almost all chronic diseases and is the basis for optimal health [7]. Alcocer-bruno et al [8] showed that poor diet, lower physical activity intensity, higher alcohol consumption (AC) and smoking were significantly associated with more HIV risk, and participants with unhealthy lifestyle were twice as likely to be infected with HIV as those with high risk. HCV infection has also been associated with unhealthy lifestyle habits such as shaver sharing, tattooing, body piercing, etc. [9]

Smoking, AC, and betel quid (BQ) chewing are very common unhealthy lifestyle habits worldwide. It has been estimated that there are approximately 1.1 billion daily smokers worldwide, and this number is expected to grow to 1.3 billion by 2025 [10]. Globally, 2.4 billion people consume alcohol [11]. Approximately 1.2–600 million people chew betel nuts, including children, to whom it is freely available for consumption [12]]. Studies have reported that smoking affects both innate and adaptive immunity [13]. Smoking is involved in many immune-produced inflammatory mediators, including pro-inflammatory cytokines and anti-inflammatory cytokines, and has a profound effect on chronic inflammation and systemic immunity [14–18]. Smoking or passive smoking has been shown to increase the risk of infections, such as Middle East Respiratory Syndrome (MERS) [19, 20]. SARS-CoV-2 MERS-CoV both belong to the same family of coronaviruses; Therefore, the relationship of smoking and COVID-19 has also attracted attention. Similarly, AC also has certain effects on the immune system, and long-term, heavy drinking has been shown to be associated with reduced production of lymphocytes and increased risk of bacterial and viral infections [21]. BQ and its ingredients, especially hydrated lime, causes continuous local stimulation of oral epithelial cells, which can cause chronic inflammation, oxidative stress, and cytokine production [22].

The study by Hamer et al. [23] indicated an unhealthy lifestyle was a risk factor for hospitalization due to COVID-19 and that lifestyle scores were strongly associated with the risk of COVID-19, with a 4.41-fold increase in those with a low lifestyle scores compared with those with the optimal lifestyle. The risk factors for COVID-19 in humans are unknown and susceptible to confounding factors; therefore, it is necessary to further study the impact of lifestyle on the susceptibility to COVID-19. Hence, the aim of our study was to analyze the impact of exercise, diet, sleep, smoking, AC, and BQ chewing on the prevention of COVID-19 by using a case control study method and thus to provide ways to guide the public to adopt a healthy lifestyle to effectively prevent COVID-19.

## Methods

### Research Setting and Inclusion and Exclusion Criteria

This case-control study included data from January 2020 to May 2021 from four hospitals (Yueyang Central Hospital; Affiliated Nanhua Hospital, University of South China; The Second People’s Hospital of Changde; Junshan District the People Hospital). Inclusion criteria for the case group were: age ≥18 years, Novel Coronavirus nucleic acid was detected by real-time fluorescence RT-PCR and the presence of normal cognitive and understanding abilities. Inclusion criteria for the control group were: Negative nucleic acid test reports of real-time fluorescence RT-PCR Novel Coronavirus within 7 days and the absence of a history of COVID-19 the presence of normal cognitive and understanding abilities. The exclusion criterion was a history of life-threatening or serious physical illness. The permission of the Ethics Committee of Hunan Cancer Hospital (SBQLL-2020-094) was obtained for the research. Patients in the case group signed written informed consent forms, whereas oral informed consent was obtained from participants of the control group.

### Data Collection

The demographic data and comorbidities of the case group were collected from the EMR system of the four hospitals, and telephone surveys were conducted with patients to obtain any missing data in the medical records. Substance usage (smoking, AC, and betel chewing) and lifestyle (diet, beverage preference, exercise status, and sleep status) were investigated. Height and weight values were obtained to calculate Body Mass Index (BMI).

The data of patients in the surgical wards of the Yueyang Central Hospital (trauma, urology, gynecology, spinal column, and pediatrics wards) or their family members were collected by trained researchers using questionnaires. A total of 1,032 individuals were surveyed. The completed questionnaires were submitted to the principal investigator for review. Finally, incomplete or invalid questionnaires were excluded and 826 valid questionnaires were retained for the analysis. Patients with COVID-19 and individuals in the control group were basically the same according to sex, marital status, place of residence, educational level, occupation, and age ±2 years, and were matched in a ratio of 1:2 to be included in the final study groups. Double input of data was performed for analysis.

### Variable Definitions and Criteria

All data were collected in accordance with previous research methods and standards.

(1) Complications: Six common complications that were diagnosed by doctors were collected from the EMR system of the hospitals; these included hypertension (HP), coronary heart disease, hyperlipidemia, renal insufficiency, and cerebral infarction. Control subjects were included by the researchers according to their previous medical history.
(2) AC: history of drinking alcohol of any type for ≥ one time per week or continuous drinking for ≥ 1 year [24].
(3) Smoker: According to WHO (1998), a smoker refers to a person who has smoked continuously or accumulatively for 6 months or more in his life [25]. In this study, data of former smokers who had quit smoking were included in the analysis.
(4) BQ chewing: history of chewing one BQ daily for more than 3 months[26].

### Statistical Analysis

Descriptive statistics were used to analyze demographic data, comorbidities, and lifestyles. Data were input into Excel, using SPSS (version 25.0; SPSS, Chicago, IL, United States). Categorical variables are expressed in terms of frequency and percentage. The chi-square test was used to compare the ratios or constituent ratios of two or more groups. Multivariate conditional logistic regression analysis was used to analyze the influence of COVID-19. Odds ratio (OR), 95% confidence interval (CI) evaluated via multivariate analysis. Statistical significance was set at *p* < 0.05.

## Results

### Demographic Characteristics of Participants

Finally, 182 COVID-19 patients and 364 participants aged 18 years and older were recruited in a 1:2 ratio in the case group and case control group, respectively. There were no statistical differences in gender, age, marriage, education level, occupation, BMI and residence between the two groups. (*p* > 0.05, Table 1). The mean age of the participants in the two groups was 47.10 ± 14.15 years and 47.19 ± 14.19 years, respectively, and most of the subjects (395/546, 72.3%) were 31–64 years old. Most patients with COVID-19 were having lower education levels, with those with high school education or below accounting for 86.3% (157/182) of the participants. Those who were retired/unemployed and engaged in self-service accounted for 22.3% (122/546) and 21.8% (119/546), respectively, of the total participants in case and control groups. Three hundred and thirty (60.4%) participants lived in urban areas and 216 (39.6%) participants resided in rural areas. BMI values ranged from 16.9 to 31.9, with a median of 23.375 (overweight, 31.0%; obese, 7.8%; and underweight, 2.6%).

**Table 1:** Univariate analysis of lifestyle between coronavirus disease 2019 patients and matched groups. (Results of the Adult COVID-19 Lifestyle Matching Study, China, 2020–2021.)

Variables	Total	Control		Case		*χ* ^2^	*p*
	N = 546 (%)	N = 364	%	N = 182	%		
Gender						0.000	1.000
Male	276 (50.5)	184	50.5	92	50.5		
Female	270 (49.5)	180	49.5	90	49.5		
Age (y)						0.117	0.943
18–30	73 (13.4)	48	13.2	25	13.7		
31–64	395 (72.3)	265	72.8	130	71.5		
≥65	78 (14.3)	51	14.0	27	14.8		
Marriage						—	0.065^a^
Unmarried	50 (9.2)	33	9.1	17	9.3		
Married	478 (87.5)	321	88.2	157	86.3		
Divorced	10 (1.8)	8	2.2	2	1.1		
Widow	8 (1.5)	2	0.5	6	3.3		
Education						—	0.082^a^
Illiteracy	19 (3.5)	12	3.4	7	3.8		
Primary school	75 (13.7)	43	11.8	32	17.6		
Middle school	220 (40.3)	138	37.9	82	45.1		
High school	134 (24.5)	98	26.9	36	19.8		
College	90 (16.5)	67	18.4	23	12.6		
Master or above	8 (1.5)	6	1.6	2	1.1		
Occupation						—	0.530^a^
Farmer	97 (17.8)	69	19.0	28	15.4		
Worker	94 (17.2)	65	17.9	29	15.9		
Office worker	91 (16.7)	61	16.8	30	16.5		
Service staff	119 (21.8)	76	20.8	43	23.6		
Retired/unemployed	122 (22.3)	76	20.8	46	25.3		
Students	18 (3.3)	12	3.3	6	3.3		
Others	5 (0.9)	5	1.4	0	0.0		
Habitation						0.000	1.000
City	330 (60.4)	220	60.4	110	60.4		
Rural	216 (39.6)	144	39.6	72	39.6		
BMI						—	0.379^a^
<18.5	14 (2.6)	12	3.3	2	1.1		
18.5–23.9	320 (58.6)	210	57.7	110	60.4		
24.0–28.0	169 (31.0)	111	30.5	58	31.9		
>28	43 (7.8)	31	8.5	12	6.6		
Underlying diseases						3.558	0.059
Yes	173 (31.7)	125	34.3	48	26.4		
No	373 (68.3)	239	65.7	134	73.6		
Hypertension						2.858	0.091
Yes	93 (17.0)	69	19.0	24	13.2		
No	453 (83.0)	295	81.0	158	86.8		
Diabetes						0.092	0.761
Yes	54 (9.9)	35	9.6	19	10.4		
No	492 (90.1)	329	90.4	163	89.6		
Cerebral infarction						9.290	0.002^b^
Yes	6 (1.1)	0	0.0	6	3.3		
No	540 (98.9)	364	100.0	176	96.7		
Coronary heart disease						1.336	0.248
Yes	19 (3.5)	15	4.1	4	2.2		
No	527 (96.5)	349	95.9	178	97.8		
Hyperlipidemia						0.134	0.715
Yes	36 (6.6)	25	6.9	11	6.0		
No	510 (93.4)	339	93.1	171	94		
Renal insufficiency						0.759	0.446
Yes	17 (3.1)	13	3.6	4	2.2		
No	529 (96.9)	351	96.4	178	97.8		
Balanced diet						0.920	0.337
Yes	401 (73.4)	272	74.7	129	70.9		
No	145 (26.6)	92	25.3	53	29.1		
Non-vegetarian diet						2.048	0.152
Yes	53 (9.7)	40	11.0	13	7.1		
No	493 (90.3)	324	89.0	169	92.9		
Vegetarian diet						5.057	0.025
Yes	53 (9.7)	28	7.7	25	13.7		
No	493 (90.3)	336	92.3	157	86.3		
Bland diet						35.821	0.000
Yes	154 (28.2)	73	20.1	81	44.5		
No	392 (71.8)	291	79.9	101	55.5		
Prefer spicy diet						0.458	0.499
Yes	111 (20.3)	77	21.2	34	18.7		
No	435 (79.7)	287	78.8	148	81.3		
Prefer sweet diet						2.776	0.096
Yes	15 (2.7)	13	3.6	2	1.1		
No	531 (97.3)	351	96.4	180	98.9		
Carbonated drinks						0.535	0.464
Yes	36 (6.6)	26	7.1	10	5.5		
No	510 (93.4)	338	92.9	172	94.5		
Fruit juice						0.091	0.763
Yes	23 (4.2)	16	4.4	7	3.8		
No	523 (95.8)	348	95.6	175	96.2		
functional beverage						0.624	0.430
Yes	10 (1.8)	5	1.4	5	2.7		
No	536 (98.2)	359	98.6	177	97.3		
Milk tea						0.000	1.000
Yes	15 (2.7)	10	2.7	5	2.7		
No	531 (97.3)	354	97.3	177	97.3		
Coffee						0.308	0.579
Yes	15 (2.7)	9	2.5	6	3.3		
No	531 (97.3)	355	97.5	176	96.7		
Chinese Tea						31.884	0.000
Yes	103 (18.9)	93	25.5	10	5.5		
No	443 (81.1)	271	74.5	172	94.5		
Smoking						4.989	0.026
Yes	137 (25.1)	102	28.0	35	19.2		
No	409 (74.9)	262	72.0	147	80.8		
Alcohol consumption						5.927	0.015
Yes	77 (14.1)	42	11.5	35	19.2		
No	469 (85.9)	322	88.5	147	80.8		
Chewing BQ						0.244	0.621
Yes	35 (6.4)	22	6.0	13	7.1		
No	511 (93.6)	342	94.0	169	92.9		
Exercise						20.900	0.000
Yes	418 (76.6)	300	82.4	118	64.8		
No	128 (23.4)	64	17.6	64	35.2		
Walk						24.621	0.000
Yes	346 (63.4)	257	70.6	89	48.9		
No	200 (36.6)	107	29.4	93	51.1		
Run						2.024	0.155
Yes	73 (13.4)	54	14.8	19	10.4		
No	473 (86.6)	310	85.2	163	89.6		
Swimming						0.000	1.000
Yes	11 (2.0)	7	1.9	4	2.2		
No	535 (98.0)	357	98.1	178	97.8		
Fitness						1.234	0.267
Yes	15 (2.7)	12	3.3	3	1.6		
No	531 (97.3)	352	96.7	179	98.4		
Dance Aerobics						0.013	0.908
Yes	40 (7.3)	27	7.4	13	7.1		
No	506 (92.7)	337	92.6	169	92.9		
Yoga						3.680	0.055^b^
Yes	10 (1.8)	10	2.7	0	0.0		
No	536 (98.2)	354	97.3	182	100.0		
Sleep pattern						7.551	0.056
before 22:00	153 (28.0)	110	30.2	43	23.6		
22:00–24:00	316 (57.9)	196	53.8	120	65.9		
after 24:00	52 (9.5)	39	10.7	13	7.1		
irregular	25 (4.6)	19	5.3	6	3.4		

a Monte Carlo *p* < 0.05.

b Correction for continuity. BMI, body mass index.

### Comorbidities of Participants in Case and Control Groups

In this study, 31.7% (173/546) of the total participants had underlying diseases, and 34.3% (125/364) of the participants in the control group had co-existing diseases, which was higher than the proportion of participants with comorbidities in the case group [26.4% (48/182)]. HP was present in 17.0% (93/546) of the total participants. There were no significant differences in proportions of participants with HP, hyperlipidemia, coronary heart disease, and renal insufficiency between case and control groups in univariate analysis. There was statistical difference in the proportion of cerebral infarction between the two groups (*p* = 0.002, Table 1).

### Dietary and Drinking Habits of Participants

Most of the participants in this study followed a balanced diet (73.4% [401/546]), and few participants followed either a nonvegetarian or vegetarian diet. The proportion of vegetarians (13.7% vs. 7.7%, *p* = 0.025) and followed bland diet [44.5% (81/182) vs. 20.1% (73/291), *p* < 0.001] between the case group and control group were statistically significant. Only 13.7% (75/546) of the total participants were regular beverage drinkers, and the proportions of participants consuming carbonated drinks, fruit juice, functional drinks, milk tea, and coffee were 6.6%, 4.2%, 1.8%, 2.7%, and 2.7%, respectively. There was no statistical significance in the type of beverages consumed between the two groups (*p* > 0.05, Table 1).

### Substance Use Among Participants in Case and Control Groups

Participants with smoking, AC, and BQ chewing behaviors accounted for 25.1% (137/546), 14.1% (77/546), and 6.4% (35/546), respectively, of the total participants. The proportion of alcohol drinkers was significantly higher in the case group than that in the control group [19.2% (35/182) vs. 11.5% (42/364), *p* = 0.015] The proportion of smokers in the case group (19.2%, 35/182) was significantly lower than that in the control group (28.0%, 102/364), and the difference was statistically significant (*p* = 0.026). There was no statistical significance in BQ chewing behavior between case and control groups (7.1% vs. 6.0%, *p* > 0.05, Table 1).

### Exercise and Sleeping Habits of Participants in Case and Control Groups

In this study, 76.6% (418/546) of the total participants had the habit of exercising, and the proportion of individuals with the habit of exercising was significantly lower in the case group than that in the control group (64.8% [118/182] vs. 82.4% [300/364], *p* < 0.001). The comparison of physical activity (PA) of walking showed that the PA of walking was significantly less among the participants in the case group compared with the individuals in the control group (48.9% [89/182] vs. 70.6% [257/364], *p* < 0.001). The proportions of participants following other exercise methods were 10.4% vs. 14.8%, 2.2% vs. 1.9%, 1.6% vs. 3.3%, 7.1% vs. 7.4%, and 0.0% vs. 2.7% in the case and control groups (*p* > 0.05). Approximately 57.9% of the participants had the habit of sleeping from 22:00 to 24:00; 28.0% and 9.5% of the individuals chose to fall asleep before 22:00 and after 24:00, respectively, and 4.6% of the participants had irregular sleeping habits. The difference was not statistically significant (*p* = 0.056, Table 1).

### Multivariate Factor Analysis

Multivariate conditional logistic regression analysis was performed with the diagnosis of COVID-19 as the dependent variable; cerebral infarction, vegetarian diet, bland diet, tea drinking, smoking, AC, exercise, walking and education level, as independent variables; *p* < 0.05 as the inclusion criterion; and *p* ≥ 0.10 as the exclusion criterion. The results showed that smokers were less likely to develop COVID-19 (OR = 0.224, 95% CI = 0.084–0.592 *p* = 0.003). Those who drank alcohol were 6.3 times more likely to develop COVID-19 compared with those who never drank alcohol (OR = 6.275, 95% CI = 2.323–16.954, *p* = 0.000). Individuals who followed a bland diet had an increased risk of COVID-19 (OR = 3.306, 95% CI = 1.891–5.778, *p* = 0.000). Tea drinking habit (OR = 0.324, 95% CI = 0.140–0.748, *p* = 0.008) and the habit of exercising of walking (OR = 0.364, 95% CI = 0.146–0.908, *p* = 0.030) were protective factors against COVID-19. Relative illiteracy with college education (OR = 0.048, 95% CI = 0.004–0.575, *p* = 0.017) and master degree OR above (OR = 0.031, 95% CI = 0.001–0.755, *p* = 0.033) was a protective factor for COVID-19 infection (Table 2).

**Table 2:** A multivariate analysis of lifestyle between coronavirus disease 2019 patients and matched groups. (Results of the Adult COVID-19 Lifestyle Matching Study, China, 2020–2021.)

Variable	B	S.E	Wald χ^2^	*p*	Estimated odds ratio	95% confidence interval
Cerebral infarction	11.421	120.296	0.009	0.924	91,255.223	0.000–2.271
Smoking	−1.498	0.497	9.081	0.003	0.224	0.084–0.592
Alcohol consumption	1.837	0.507	13.117	0.000	6.275	2.323–16.954
Vegetarian diet	0.170	0.474	0.128	0.720	1.185	0.468–3.004
Bland diet	1.196	0.285	17.618	0.000	3.306	1.891–5.778
Drinking tea	−1.128	0.427	6.969	0.008	0.324	0.140–0.748
Exercise	0.131	0.493	0.071	0.790	1.140	0.434–2.997
Exercise -Walking	−1.012	0.467	4.694	0.030	0.364	0.146–0.908
illiteracy			14.066	0.015		
Primary school	−0.381	1.066	0.128	0.721	0.683	0.085–5.517
Middle school	−1.182	1.137	1.081	0.299	0.307	0.033–2.848
High school	−2.242	1.177	3.631	0.057	0.106	0.011–1.066
College	−3.035	1.266	5.748	0.017	0.048	0.004–0.575
Master or above	−3.459	1.621	4.552	0.033	0.031	0.001–0.755

## Discussion

We performed a matching study according to educational background, occupation, place of residence, marital status, sex, and age to elucidate the relationship between lifestyle and the risk of COVID-19 in adults. There was no difference after statistical analysis (*p*<0.05).

### Physical Activity Is a Protective Factor Against COVID-19

This study revealed that 76.6% of the total participants performed regular physical activities, especially walking (63.4%). Usually, Chinese adults indulge in daily exercises to a greater extent. The number of people who exercised, especially of those who walked, was significantly reduced in the case group compared with the control group (Table 1). Studies have reported that routine PA has a protective effect on the immune system, can enhance the immune response to viral antigens, and reduces the incidence of viral infections [4, 27]. Furthermore, PA can increase the endurance of respiratory muscles or improve the immune response to respiratory viral antigens, and thus prevent respiratory viral infections [28]. Moderate-intensity exercise has a protective effect on respiratory viral infections, while high-intensity exercise may increase the risk of infections [29]. Prolonged and intense exercise can considerably enhance the immune response of the body; however, it can also facilitate infectious processes by viruses, leading to more pathological harm [30]. Simpson et al. [31] had reported that an appropriate extent of PA was beneficial to health and immune functions, and vigorous exercise might lead to an increased risk of disease. Da Silveira et al [32]. confirmed that physical exercise could improve immune functions and prevent COVID-19 in humans. This study suggests that walking may have a potential protective effect against COVID-19 infection. This may be related to the fact that walking can enhance human immunity, and the wide outdoor environment, away from crowded indoor environment, can reduce the chance of close contact with others, thus reducing the risk of infection. Only walking was included as a variable in the multivariate conditional regression analysis in our study; however, other forms of exercise were also found to be beneficial. This may be related to the “J-shaped” hypothesis of physical exercise [34]. Therefore, sedentary behavior should be avoided [33]. Although any PA is beneficial, it should be noted that exercise should be moderate and tolerable, and prolonged and strenuous exercise should be avoided [34]. Compared to other forms of exercise, walking is an easy, free, convenient exercise that can help people fight against COVID-19.

### Relationship Between Underlying Diseases and Bland Diet, and COVID-19

Our study suggested that people who eat a bland diet are at a higher risk of COVID-19. Although multivariate analysis in this study demonstrated that comorbidities were not risk factors for COVID-19. However, we noticed that people with comorbidities followed a bland diet mostly. Approximately 28.2% (154/546) of the total participants and 34.1% (59/173) of the patients with comorbidities followed a bland diet (*p* = 0.037). In particular, 58.8% (10/17) of the patients with nephropathy had followed a bland diet (*p* = 0.007). However, this study failed to confirm the relationship between underlying diseases and COVID-19. Nutrition can affect the immune system and determine the risk and severity of infections [35]. It has been reported that some nutrients may be associated with the prevention and treatment of COVID-19. These include vitamins such as vitamin A, B, C, D, and E; minerals such as iron, zinc, and selenium; fiber; and essential fatty acids [36]. WHO stress the balanced diet can maintain a strong immune system and avoid or minimize the incidence of chronic and infectious diseases. It has been recommended to follow daily dietary recommendations by WHO to enhance immunity for improved protection against COVID-19 and chronic diseases [37].

### Tea Drinking Is a COVID-Protective Factor

**FIGURE 1:**
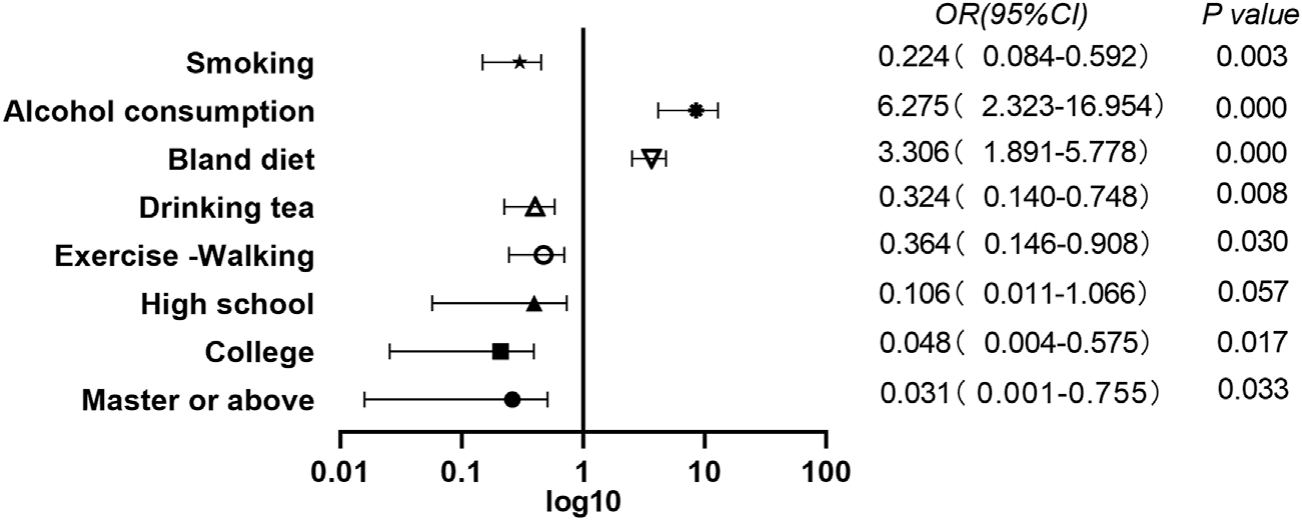
A multivariate analysis of lifestyle between coronavirus disease 2019 patients and matched groups.(Results of the Adult COVID-19 Lifestyle Matching Study, China, 2020–2021.)

Tea and its bioactive polyphenols have been shown to have many beneficial effects on health [38]]. Results of this study showed that tea drinkers had a significantly decreased COVID-19 risk (OR = 0.324, 95% CI = 0.140–0.748, *p* = 0.008). This may be related to the antiviral effect of polyphenols in tea [39]. Tea is rich in antioxidants, complex polyphenols, micronutrients, and vitamins. Studies [40] have shown that polyphenols and micronutrients can enhance immunity, which may have strong significance in preventing COVID-19. EGCG (epigallocatechin-3-gallate) in green tea extract has been shown to have a significant inhibitory effect on SARS-CoV-2 and other coronavirus infections [41]. EGCG can inhibit the expression of angiotensin-converting enzyme-2 (ACE-2) and transmembrane protease serine 2 (TMPRSS 2) on the cell surface by activating NRF 2, thus inhibiting SARS-CoV-2 infection. Meanwhile, EGCG may also have a protective effect on oxidative stress and cytokine storm started by SARS-CoV-2 [42, 43]. Moreover, catechins have anti-inflammatory and potential therapeutic effects against COVID-19 [44]. The micronutrients, vitamins in tea have also been proved to be beneficial in blocking the infection of COVID-19 and reducing COVID-19 risks [45]. Studies have shown that drinking three cups of tea daily not only has no adverse effects on human health but also is beneficial [46]]. Therefore, tea is recommended as a healthy beverage to help prevent COVID-19. Figure 1.

### Smoking and the Risk of COVID-19

The relationship between smoking and susceptibility to COVID-19 has been the focus of scientific debate. Existing studies have shown that the ACE-2 receptor is the entry point of SARS-CoV-2 into host cells and SARS-CoV-2 is more likely to bind and infect human cells than other coronaviruses [47]. However, smoking has been reported to increase the expression of ACE-2 receptors; therefore, smoking is thought to increase the risk of COVID-19 [48, 49]. Contrary to our prediction, our study showed that smokers had a significantly reduced risk of COVID-19 compared with nonsmokers (OR = 0.224, 95% CI = 0.084–0.592 *p* = 0.003). This is consistent with the results of the systematic review by Simons D et al [50]. Several other studies have also reported COVID-19 patients had lower smokers rate than those the public [51, 52]. The smoker rate of COVID-19 patients in China accounts for only one-fourth of the proportion of smokers among the public in China [53, 54]. This study showed COVID-19 patients smoking rate was 19.2% (35/182), which also was significantly lower than the 28.0% (102/364) of the control group. Results from a study involving 175 countries also showed that globally, the percentage of smokers in a population is inversely correlated with the incidence of COVID-19 [55]. Some studies suggest that this may be related to nicotine, a component of tobacco [53, 56]. Nicotine is a cholinergic agonist, which has anti-inflammatory effects and can regulate the immune response of the body [57, 58]. Nicotine suppresses the production of pro-inflammatory cytokines in smokers may render their immune responses more tolerable than those in patients who have never smoked [57, 59]. Moreover, nitric oxide (NO) generated during smoking is involved in maintaining airway dilation and filtration before entering the lungs. However, NO has been shown to inhibit the replication and entry of SARS-CoV-2 into body cells [60]. While these mechanisms may indicate the potential therapeutic effects of nicotine, tobacco smoke is known to be a complex mixture of over 5,000 chemicals, carcinogens, or toxins [61]. Smoking not only affects one’s overall health but also damages almost every organ in the body [62, 63]. Even though smokers may have lower incidence rates of COVID-19 than those in normal people, the adverse health effects of smoking surpass the benefits [56]. Therefore, the WHO advises the public to quit smoking and to be cautious about smoking as a method of protection against COVID-19.

### AC Significantly Increases the Risk of COVID-19

Our study found that AC also increased the risk of COVID-19, and the risk of COVID-19 in those who drank alcohol was 6.3 times higher than that in those who never drank alcohol (OR = 6.275, 95% CI = 2.323–16.954, *p* = 0.000), consistent with the results of Bailey et al [64]. There is also evidence that AC increases the risk of bacterial pneumonia, tuberculosis, respiratory syncytial virus (RSV) infection, and acute respiratory distress syndrome (ARDS) [65]. This may be related to the damage to the immune system caused by alcohol [66]. The immune system protects the host against pathogens and unwanted immune responses to the body, including innate and adaptive immunity. Alcohol has been shown to interfere with both types of immunity [67, 68]. Alcohol also changes the balance and interaction between the immune system and the microbiome of the host. There is evidence that any level of AC is not good for your health and that even one drink can damage your health [69, 70]. However, Ahmed et al. [71] reported that during the period of social isolation during the COVID-19 outbreak, the incidence of alcohol use disorders (AUDs) in Hubei province increased 10 times more than that in other provinces with fewer restrictions. In Britain, a study reported that one in five people had consumed increased amounts of alcohol since incarceration [72]]. It is important to recognize that AC increases the risk of COVID-19, and people should be encouraged to avoid or reduce AC to prevent COVID-19.

### College Degree or Above is a Protective Factor for COVID-19 Infection

Our case-control study suggested that education levels were associated with COVID-19 susceptibility. College degree or above relative illiteracy is COVID-19 protective factors of infection; this is consistent with the findings of Hawkins et al. [73]. People with higher education may pay more attention to the epidemic situation and have stronger protection awareness, while those with lower education may have a lowered ability to acquire health knowledge and take protective measures. Zhong et al. [74] showed that there was a significant positive correlation between education level and COVID-19 knowledge score; the higher the COVID-19 knowledge score, the lower the likelihood of negative attitudes and potentially dangerous practices toward COVID-19 prevalence.

### Limitations

First, in this study, a convenience sampling method was adopted for the matched samples of patients or their family members from the surgical wards of a general hospital. Second, although cerebral infarction was included in the multivariate regression analysis, there were only six cases of cerebral infarction in the case group; therefore, the sample size was too small. These two factors may have caused the bias of the respective results. Future researches based on these limitations are required.

### Conclusion

After controlling for education, occupation, place of residence, marital status, sex and age, our study found that physical activity, particularly regular walking and drinking more tea, college degree or above was associated with a reduced risk of COVID-19 infection, while drinking any type of alcohol significantly increased the risk of COVID-19. Although this case-control study showed that smokers had a lower rate of incidence of COVID-19 than that among non-smokers, the underlying mechanism could not be elucidated; therefore, further studies should be conducted. Previous studies have confirmed that smoking is harmful; therefore, smoking should be discouraged and smokers should be encouraged to quit smoking.
